# Heat-Inactivated *Lactiplantibacillus plantarum* (RSB11) as a Multifunctional Postbiotic: Anti-Inflammatory Effects Compared with Live RSB11 Across Multiple Human Cell Models

**DOI:** 10.3390/cells15191736

**Published:** 2026-09-23

**Authors:** Teodora Nicola, Tanmaya Madhvacharyula, Ajay Ashok, Aryan Mandot, Iman Abdelgawad, Ryaan Singh, Kara Siedman, Youfeng Yang, Namasivayam Ambalavanan, Charitharth Vivek Lal

**Affiliations:** 1 Division of Neonatology, Department of Pediatrics, University of Alabama at Birmingham, Birmingham, AL 35233, USA; 2 Schepens Eye Research Institute of Massachusetts Eye and Ear Infirmary, Harvard Medical School, Boston, MA 02114, USA; 3 Lung Health Center, University of Alabama at Birmingham, Birmingham, AL 35233, USA; 4 Marnix Heersink Institute of Biomedical Innovation, University of Alabama at Birmingham, Birmingham, AL 35233, USA

**Keywords:** *Lactiplantibacillus plantarum*, RSB11, postbiotic, heat inactivation, inflammation, MMP-9, cytokines, gut–ovarian axis

## Abstract

**Highlights:**

**What are the main findings?**

- Heat-inactivated RSB11 (RSB11-HI) retained the broad anti-inflammatory activity of its live counterpart, suppressing MMP-9, TNF-α, IL-6, and IL-1β responses across diverse human cell models representing the gut, lung, ovary, bone, kidney, and liver, with the two forms compared directly at each of these endpoints.
- Beyond inflammatory modulation, RSB11-HI increased β-glucuronidase-associated and estradiol measurements relative to untreated controls in a gut–ovarian conditioned-medium model, suggesting additional effects on microbiome–endocrine signaling.

**What are the implications of the main findings?**

- Preservation of RSB11 bioactivity after heat inactivation demonstrates that microbial viability is not required for its observed anti-inflammatory effects and supports development of RSB11-HI as a candidate postbiotic.
- Retaining biological activity in an inactivated preparation may broaden the formulation and application potential of RSB11 while providing a foundation for further mechanistic and preclinical investigation.

**Abstract:**

The clinical potential of probiotics is increasingly recognized; however, dependence on microbial viability, formulation and storage constraints, and safety considerations in vulnerable populations can limit their broader application. Postbiotics offer an alternative by providing inanimate microbial preparations with retained biological activity, yet whether heat inactivation preserves the functional effects of probiotic-derived candidates remains an important question. Here, we compare *Lactiplantibacillus plantarum* RSB11 in its live (RSB11 life) and heat-inactivated (RSB11-HI) forms across human cell models representing the gut, lung, ovary, bone, kidney, and liver. Following inflammatory challenge with lipopolysaccharide or *Escherichia coli*, we assessed MMP-9 expression and the pro-inflammatory cytokines TNF-α, IL-6, and IL-1β. Both RSB11 life and RSB11-HI broadly suppressed inflammatory responses, with RSB11-HI maintaining effects comparable to the live preparation across the MMP-9 and cytokine endpoints, where the two forms were tested side by side, and demonstrating greater suppression in selected experimental contexts. Additionally, RSB11-HI increased β-glucuronidase and estradiol levels relative to untreated controls in a gut–ovarian conditioned-medium model, suggesting effects extending beyond inflammatory modulation; a live comparator was not included in the β-glucuronidase measurements, so this effect is not established as specific to the inactivated form. Collectively, these findings demonstrate that heat inactivation preserves key biological functions of RSB11 and support RSB11-HI as a multifunctional candidate postbiotic with potential for expanded formulation and application.

## 1. Introduction

Chronic, low-grade inflammation underlies the pathogenesis of a wide spectrum of human diseases, including gastrointestinal disorders, chronic respiratory disease, metabolic syndromes, reproductive dysfunction, liver fibrosis, kidney disease, and neurodegeneration. Central to these processes are inflammatory mediators such as tumor necrosis factor-α (TNF-α) [1], interleukin-6 (IL-6) [2], interleukin-1β (IL-1β) [3], and matrix metalloproteinase-9 (MMP-9) [4], which amplify cytokine cascades, promote extracellular matrix remodeling, and disrupt tissue homeostasis [5]. While pharmacological agents targeting these pathways exist, they are often costly, associated with systemic side effects, or limited to specific disease indications. This has spurred growing interest in microbiome-based interventions as safe, broadly acting strategies for dampening inflammation and restoring host–microbiome balance [6,7,8].

Probiotics have been extensively studied in this context and have shown immunomodulatory effects in both preclinical and clinical settings [7,9,10,11]. However, their widespread adoption as therapeutics has been hindered by major practical and biological constraints [12,13]. Chief among these is the reliance on viability since live microorganisms require precise storage conditions [14], are vulnerable to degradation when mixed with supplemental compounds [15], and exhibit unpredictable survival within the gastrointestinal tract. These issues surrounding the use of live forms of microorganisms bring variability in efficacy across batches and formulations, undermining reproducibility and reliability. Moreover, safety concerns have been raised regarding the administration of live bacteria in immunocompromised, critically ill, or neonatal populations, where translocation or overgrowth could pose risks [16,17,18]. Thus, while probiotics remain conceptually attractive, the requirement for microbial viability limits their translational feasibility and scalability.

Postbiotics, defined as preparations of inanimate microorganisms and/or their bioactive components that confer health benefits to the host have emerged as a solution to these limitations [19,20]. By decoupling efficacy from viability, postbiotics offer enhanced stability, reproducibility, and safety. They can be stored without refrigeration, formulated into diverse delivery systems, and used in populations where live organisms are contraindicated [21]. Despite this promise, a key unresolved question has been whether heat inactivation compromises the function and efficacy of these formulations. Exploring the efficacy of heat-inactivated (HI) formulations is therefore critical. If shown to retain the functional activity of live probiotics, HI preparations could overcome barriers that currently restrict probiotic application. They could combine biological activity with the formulation advantages of an inactivated preparation, potentially enabling broader applications without dependence on microbial viability. Demonstrating preservation of biological activity following heat inactivation therefore represents an important advance in the development of microbiome-based interventions.

In the present study, we address this critical gap by comparing the effects of a candidate postbiotic, RSB11, in both its live (RSB11 life) and heat-inactivated (RSB11-HI) forms [11]. Using a panel of human epithelial and non-epithelial cell models representing the gut [4,22], lung [23], ovary [24], bone [25], kidney [26], and liver [27], we examined their ability to suppress MMP-9, TNF-α, IL-6, and IL1β expression in response to inflammatory challenge with *E. coli* or lipopolysaccharide (LPS). We further assessed metabolic cross-talk by evaluating β-glucuronidase levels and estrogen modulation along the gut–ovarian axis. By deconjugating glucuronidated estrogens, β-glucuronidase facilitates their reactivation and recirculation, thereby influencing local and systemic estrogen bioavailability [28]. Assessing β-glucuronidase levels provides functional insight into how RSB11-HI may restore microbial endocrine balance and modulate estrogen-dependent inflammatory responses in ovarian and gut epithelial systems. By demonstrating that heat-inactivated RSB11 retains full immunomodulatory efficacy, this work establishes a foundation for the development of RSB11-HI as a next-generation postbiotic therapeutic for inflammation-driven disorders. RSB11 is a *Lactiplantibacillus plantarum* strain characterized in our earlier work as a component of a lactobacilli-based inhaled live biotherapeutic product, in which it attenuated MMP-9/PGP-driven neutrophilic inflammation, downregulated NF-κB signaling in human bronchial epithelial cells, and improved the lung structure and function in murine models of chronic lung disease. The live and heat-inactivated preparations compared here derive from this same characterized strain, allowing the contribution of viability to its biological activity to be assessed directly.

## 2. Materials and Methods

### 2.1. Reagents and Materials

Lipopolysaccharide (LPS) [29] and *E. coli* (10^7^ CFU/mL) [30] were used as inflammatory stimuli. Unless specified, molecular biology reagents (RNeasy Mini Kit (Qiagen, Hilden, Germany), iScript™ cDNA synthesis kit (Bio-Rad Laboratories, Hercules, CA, USA), TaqMan master mix (Thermo Fisher Scientific, Waltham, MA, USA) and ELISA kits (R&D Systems, Minneapolis, MN, USA) were from standard vendors. Microplates were read on a BioTek Epoch reader.

### 2.2. Cell Lines and Culture Conditions

Human 16HBE14o− bronchial epithelial cells (MilliporeSigma, Burlington, MA, USA; SCC150), Caco-2/TC-7 human colon adenocarcinoma cells (MilliporeSigma, USA; SCC209), and BG-1 human ovarian adenocarcinoma cells (MilliporeSigma, USA; SCC415) were obtained commercially. MG-63 human osteosarcoma cells (ATCC CRL-1427), HepG2 human hepatocellular carcinoma cells (ATCC HB-8065), and A-498 human renal carcinoma cells (ATCC HTB-44) were obtained from the American Type Culture Collection (ATCC, Manassas, VA, USA). 16HBE14o− cells were cultured according to the manufacturer’s recommendations at 37 °C, 5% CO_2_, with medium changes every 2–3 days. Caco-2/TC-7 cells were maintained in DMEM with 10% FBS, 1% penicillin–streptomycin, and 1% non-essential amino acids at 37 °C, 5% CO_2_. MG-63 and BG-1 cells were cultured in EMEM with 10% FBS and 1% antibiotic–antimycotic. HepG2 cells were maintained in Eagle’s Minimum Essential Medium (EMEM; Gibco, Thermo Fisher Scientific, USA), formulated with Earle’s balanced salts, non-essential amino acids and 2 mM L-glutamine, and further supplemented with 10% heat-inactivated FBS, 1% non-essential amino acids, and 1% penicillin–streptomycin. A-498 cells were grown in RPMI-1640 with 10% FBS and 1% antibiotic–antimycotic. Cells were sub-cultured at ~70–90% confluence according to each cell line’s standard practice. All basal media, sera, antibiotics, and supplements were obtained from Gibco/Thermo Fisher Scientific (Waltham, MA, USA); LPS was from Sigma-Aldrich (St. Louis, MO, USA); DuoSet ELISA kits were from R&D Systems (Minneapolis, MN, USA); and RNeasy Mini Kits were from Qiagen (Hilden, Germany). The cell lines and measured endpoints are summarized in Table 1.

### 2.3. Availability of the Heat-Inactivated Postbiotic (RSB11-HI)

The live and heat-inactivated *Lactiplantibacillus plantarum* RSB11 strain was kindly provided by Resbiotic Nutrition Inc., Birmingham, AL, USA (www.resbiotic.com) [31]. Several concentrations were tested (optimized in earlier work), and a working concentration of 3 mg/mL was sufficient to produce the desired experimental outcome [11,32]. The full strain designation is *Lactiplantibacillus plantarum* RSB11 HI, deposited under accession number CECT 4528; species identity was established by 16S rRNA gene sequencing and strain identity by pulsed-field gel electrophoresis (PFGE). The heat-inactivated material is supplied as a freeze-dried powder. The commercial ingredient consists of heat-inactivated RSB11 together with microcrystalline cellulose as an excipient and carrier; the manufacturer’s documentation does not state whether cell lysates, fermentation supernatant, secreted metabolites, or residual fermentation medium are also present, and we therefore cannot exclude their contribution to the activity reported here. The product is standardized by cell count, rather than by dry weight, to a specification of at least 4.00 × 10^11^ inactivated cells per gram; the lot used throughout this study assayed at 5.10 × 10^11^ cells per gram. The 3 mg/mL working concentration therefore delivers approximately 1.5 × 10^9^ inactivated cells per mL, with the remainder of the mass accounted for by the cellulose carrier. Residual viable organisms are quantified by the manufacturer using method QC-AM-001 against an acceptance criterion of fewer than 100,000 CFU/g. The lot used here (finished lot FPU2500161, mix lot MXU2500057, product code K001144, produced 22 March 2025, best before February 2026) returned 9000 CFU/g, so the preparation is not sterile; at the working concentration, this corresponds to roughly 27 CFU/mL, or about one culturable organism for every 5 × 10^7^ inactivated cells. This residual fraction is far too small to account for the effects reported, but we state it explicitly rather than describe the preparation as devoid of viable bacteria. Heat inactivation was performed by the manufacturer as a controlled thermal treatment of the harvested biomass, selected to abolish viability while preserving cell-envelope integrity. The material is produced in an FSSC 22000-certified facility and released against microbiological specifications for pathogens and indicator organisms, and against heavy metal limits of arsenic below 1.5 ppm, cadmium below 0.5 ppm, lead below 0.5 ppm, and mercury below 1.5 ppm, determined by ICP-MS. The Certificate of Analysis and Technical Data Sheet for this lot are provided as Supplementary File S1. The freeze-dried powders, both live and heat-inactivated, were stored at −80 °C to ensure the long-term stability of the biological material. Frozen storage at −80 °C was adopted here solely as a laboratory measure to hold a single lot unchanged across the full series of experiments and to keep the live comparator arm viable. The manufacturer does not require it; the recommended conditions are −18 °C or below for an 18-month best-before period, or 8 °C or below for a 12-month period, and the lot used here was within its best-before date throughout the study. Freedom from cold-chain dependence remains one of the practical advantages of the inactivated preparation. In this study, the term “heat-inactivated” refers to a heat-treated preparation produced under controlled thermal conditions to ensure safety and functional stability. The thermal process, its in-process controls and the release testing applied to the lot used here are documented in Supplementary File S1 and in the accompanying Certificate of Analysis, which are provided with this revision. Further information on the procedure and certification or analysis is provided in the Supplementary File S1.

### 2.4. Inflammatory Stimulation and Treatment Paradigms

Cells were seeded in 24-well plates and grown to ~80% confluence. Inflammation was induced with LPS (1 µg/mL) or by *E. coli* (10^7^ CFU/mL) incubation for 4 h. For each cell line, the following groups were run in parallel: untreated control; LPS/*E. coli* only; RSB11 life only, RSB11-HI only; LPS/*E. coli* + RSB11 life and LPS/*E. coli* + RSB11-HI. For the gut-ovarian axis the supernatants from previously treated Caco-2 cells were collected and used for 4 h treatment of BG1 ovarian cells while an equal volume of fresh EMEM media was added. The inflammatory stimulus was chosen according to the exposure each tissue encounters physiologically. Caco-2 and 16HBE14o—cell model mucosal barriers that meet whole bacteria at their apical surface—were therefore challenged with intact *E. coli*, whereas BG-1, MG-63, A-498, and HepG2 cells represent internal tissues exposed to circulating endotoxin rather than to intact luminal organisms and were challenged with purified LPS. Because intact *E. coli* engages multiple pattern-recognition receptors while LPS acts predominantly through TLR4, all comparisons in this study are made within a given cell model between challenged and treated conditions; no quantitative comparison is drawn across models receiving different stimuli. For the same reason, stimulus-free RSB11 life-only and RSB11-HI-only groups were included in the barrier models, where direct apical exposure to a whole-bacterial preparation made it necessary to verify that neither preparation was itself pro-inflammatory. Having established in those models that neither altered basal MMP-9 expression, these groups were not repeated in all-tissue models, in which the preparations were tested. This accounts for the difference in group structure between figures.

### 2.5. RNA Isolation and Quantitative PCR

Total RNA was isolated (RNeasy Mini Kit, Qiagen) and reverse-transcribed (iScript™, Bio-Rad). qPCR was performed using TaqMan Master Mix (Thermo Fisher Scientific) on a QuantStudio 3 system per the manufacturer’s instructions. Relative expression was calculated by the 2^−ΔΔCt^ method. Quantitative real-time PCR (qPCR) was performed using primer probes for human MMP-9 (Hs00957562_m1, Thermo Fisher) per the manufacturer’s instructions. qPCR was performed using an initial 10 min denaturation period at 95 °C followed by 40 cycles of 15 s at 95 °C and 1 min annealing and extension at 60 °C. Expression levels of MMP-9 were normalized to 18 S RNA (Thermo Fisher Scientific) while running simultaneously in each reaction. TaqMan is a probe-based assay that offers high specificity for target nucleic acids.

### 2.6. Analysis of TNF-α, IL6 and IL1β by ELISA

Cell culture supernatants were collected and stored at −80 °C. Cell pellets were harvested in RIPA buffer containing protease inhibitors after the treatment period, clarified to remove debris, and analyzed using human DuoSet TNF-α, IL6, and IL1β ELISA (R&D Systems) following the manufacturer’s protocol; absorbance was read at 450 nm on a BioTek Epoch. Data were normalized to a standard curve.

### 2.7. β-Glucuronidase (GUSB) Levels

GUSB levels in supernatants were quantified using a commercial β-glucuronidase ELISA kit (EH43RB-Thermo Scientific) according to the manufacturer’s instructions; values were normalized to a standard curve.

### 2.8. E2 Activity

Estrogen was measured in supernatants by a human estradiol E2 ELISA kit following the manufacturer’s protocol (KAQ0622-Invitrogen, Carlsbad, CA, USA); values were normalized to a standard curve.

### 2.9. Statistical Analysis

All experiments were performed with technical replicates and independently repeated at least three times. Technical replicates were averaged within each independent experiment, and *n* represents the number of independent experiments. Data were analyzed using GraphPad Prism 9 (GraphPad Software, San Diego, CA, USA) and are presented as mean ± SEM. Statistical significance was assessed using one-way analysis of variance (ANOVA) followed by Tukey’s post hoc multiple-comparisons test. A *p*-value < 0.05 was considered statistically significant. Where *n* values varied, this reflected the exclusion of outliers prior to statistical analysis using the ROUT method (Q = 1%) implemented in GraphPad Prism. The procedure was applied uniformly to every experimental group before any group comparison was performed and without reference to the outcome of those comparisons. No more than one value was removed from any group, and the direction and statistical significance of every reported effect were unchanged when the full uncensored dataset was reanalyzed.

## 3. Results

### 3.1. RSB11 Suppresses MMP-9 Expression Across Multiple Cell Models

To determine whether RSB11 in both its live and heat-inactivated (HI) form modulates inflammatory protease activity, we measured MMP-9 expression in epithelial and non-epithelial cell types subjected to inflammatory injury. LPS stimulation (1 µg/mL, 4 h) or *E. coli* exposure (10^7^ CFU/mL) markedly increased *MMP-9* mRNA in gut (Caco-2) (Figure 1A), lung (HBE) (Figure 1B), ovary (BG1) (Figure 1C), bone (osteoblast MG-63) (Figure 1D), kidney (A-498) (Figure 1E), and liver (HepG2) cells (Figure 1F), confirming the activation of inflammatory pathways in each model. Treatment with RSB11 at 3 mg/mL significantly suppressed MMP-9 expression across all cell lines tested. Importantly, the extent of suppression was indistinguishable between the live (RSB11 life) and heat-inactivated (RSB11-HI) forms, with both preparations reducing LPS-induced MMP-9 to near-baseline levels. Both the live and HI formulations did not show any significant alterations in MMP-9 expression when treated in the absence of insults (LPS and *E. coli*) demonstrating their biosafety in these cell models. These findings clearly demonstrate that the ability of RSB11 to attenuate protease expression is not dependent on microbial viability.

### 3.2. RSB11 Retains Robust TNF-α Suppression Following Heat Inactivation

Parallel analysis of cytokine production revealed that LPS and *E. coli* injury induced robust secretion of TNF-α, a pro-inflammatory cytokine, in all cell types. Both RSB11 life and RSB11-HI significantly reduced TNF-α levels, with reductions ranging from 40 to 70% depending on the cell type. Once again, no statistically significant difference was observed between live and inactivated preparations. The anti-inflammatory effects were consistently retained across epithelial (Caco-2 (Figure 2A), HBE (Figure 2B), BG1 (Figure 2C)) and non-epithelial (HepG2 (Figure 2E), A-498 (Figure 2H), MG63 osteoblasts (Figure 2D)) cell models in both the protein from the cell pellet (Figure 2A–E) and supernatant (Figure 2F–H), highlighting a broad anti-inflammatory profile retained after heat inactivation.

### 3.3. RSB11 and RSB11-HI Attenuate IL-6 Responses Across Multiple Cell Types

RSB11 life and its heat-inactivated form (RSB11-HI) demonstrated differential regulation of IL-6 across cell types. In Caco-2 and BG1 cells, neither RSB11 life nor RSB11-HI significantly suppressed IL-6 levels, intracellularly. However, in supernatants, only RSB11-HI significantly reduced IL-6 levels in BG1 cells (Figure 3A,C). In contrast, HBE cells exhibited a strong LPS-driven IL-6 increase, which was significantly reduced by both RSB11 life and RSB11-HI in cells (Figure 3B).

The most pronounced effects were observed in MG63 and A498 cells. LPS markedly elevated IL-6 levels both intracellularly and in culture supernatants. Treatment with either RSB11 life or RSB11-HI significantly reduced IL-6 expression and secretion, with RSB11-HI consistently exerting stronger suppression in MG63 cells. HepG2 cells also showed significant IL-6 reduction in cell pellets after treatment, though the supernatant levels were only modestly decreased. Collectively, these results indicate that both RSB11 life and RSB11-HI attenuate LPS-induced IL-6 responses in a cell-type-dependent manner, with robust suppression in osteoblast-like (MG63) and renal carcinoma (A498) cells (Figure 3D–J).

### 3.4. RSB11 Formulations Mitigate IL-1β-Driven Inflammatory Responses

Similar to IL-6, responses of IL-1β, an inflammatory cytokine, varied among cell types. In Caco-2 cells, HBE cells, BG1 cells, and the supernatant, RSB11 life mitigated IL-1β levels; however, RSB11-HI produced significantly higher suppression of intracellular IL-1β in HBE and A498 cells (Figure 4A–C,G). However, in MG63, A498, and HepG2 cells, both forms of RSB11 significantly reduced LPS-induced IL-1β expression both intracellularly and secreted. A498 cells showed the strongest reduction, with IL-1β levels nearly normalized to control values following postbiotic treatment in cells, with the heat-inactivated form showing the maximum efficacy in doing so (Figure 4D–J). These findings indicate that both live and heat-inactivated RSB11 effectively blunt LPS-driven IL-1β responses across multiple cell types with the heat inactivated form being the most potent consistently.

### 3.5. RSB11-HI Enhances β-Glucuronidase Levels and Modulates Estrogen Levels Along the Gut–Ovarian Axis

To assess whether RSB11-HI influences metabolic pathways relevant to hormonal regulation, we quantified β-glucuronidase (β-GUS) levels and estrogen (E2) levels in gut–ovary co-culture models. Treatment with RSB11-HI significantly increased β-glucuronidase levels compared to control conditions (Figure 5A). Figure 5A,B compare RSB11-HI with untreated control conditions only and do not include a parallel RSB11 life arm. These data therefore show that RSB11-HI raises β-GUS levels relative to the control but do not establish that the effect is specific to, or greater than, that of the live preparation. In gut supernatants, RSB11-HI nearly doubled β-GUS levels, while in the ovary–gut axis model, RSB11-HI also induced a robust increase in β-GUS compared to untreated controls (Figure 5B).

This enzymatic upregulation corresponded with a significant rise in estrogen production. In ovary–gut co-cultures, RSB11-HI treatment resulted in a ~3-fold increase in E2 levels compared to control and RSB11 life treatments, indicating a stronger effect of the heat-inactivated form (Figure 5C). Notably, exogenous estrogen supplementation validated the system, producing the highest E2 levels. These findings suggest that RSB11-HI not only suppresses inflammatory cytokines but also modulates metabolic-hormonal cross-talk along the gut–ovarian axis by enhancing β-glucuronidase levels and promoting estrogen production.

## 4. Discussion

In this study, we applied the “gut-X axis” concept to outline the complex communication pathways between the gut and other distant organs (lung, liver, ovary, bone, kidney) and discussed our latest findings related to the effects of RSB11HI postbiotic on reducing inflammation and its implications in the gut-ovarian axis [33,34,35]. This bi-directional communication is mediated by factors like immune cells, hormones, and microbial metabolites, which can travel from the gut to these organs and influence their metabolic status, immune responses, and overall homeostasis [35,36].

Previously, we published the gut–lung axis where we tested an inhaled live biotherapeutic (LBP) using active Lactobacillus strains in in vitro and in vivo models of chronic lung diseases like BPD and COPD. The Lactobacillus-based LBP is effective in improving lung structure and function, mitigating neutrophil influx, and reducing a broad swath of pro-inflammatory markers in these models of chronic pulmonary disease via the MMP-9/PGP (matrix metalloproteinase/proline-glycine-proline) pathway [11,32].

In this in vitro study, we extend our research to other organs and compare the live biotherapeutic (RSB11 life) to its heat-inactivated form (RSB11-HI), a Resbiotic Nutrition Inc. proprietary form [11,31]. Understanding these gut-X-axes opens new avenues for treatment, such as using postbiotics or dietary interventions to target the gut microbiota and improve conditions in other organs [34,35,37]. The therapeutic potential of RSB11-HI may have forthcoming clinical relevance in various diseases, including metabolic disorders, mental health conditions, and autoimmune diseases by potentially reducing systemic inflammation.

Current data provides compelling evidence that the postbiotic RSB11 retains potent immunomodulatory and metabolic functions even after heat inactivation. Both live RSB11 and its heat-inactivated counterpart (RSB11-HI) significantly attenuated pro-inflammatory cytokines, including TNF-α, MMP-9, IL-6, and IL-1β, across diverse cell types representing gut, lung, ovary, bone, kidney, and liver systems. Importantly, RSB11-HI retained the robust anti-inflammatory effects of the live form and, in some contexts, demonstrated superior efficacy. Beyond immunomodulation, RSB11-HI increased β-glucuronidase levels and estrogen production along the gut–ovarian axis, expanding its functional profile to include host–microbiome hormonal cross-talk. Because the β-glucuronidase measurements were made against untreated controls without a parallel RSB11 life arm, we do not claim this activity as unique to the heat-inactivated form; whether it distinguishes RSB11-HI from RSB11 life requires a direct side-by-side comparison. These findings reframe the paradigm of probiotic therapeutics by showing that microbial viability is not essential for therapeutic benefit, and that structural or metabolite-retained components of postbiotics may provide a safer, more versatile alternative. This study employed a heat-treated preparation of RSB11 produced under controlled thermal conditions to ensure consistency and stability. The similar magnitude of anti-inflammatory activity observed between the live and heat-treated forms across the MMP-9 and cytokine endpoints highlights the robustness and reproducibility of the RSB11 formulation.

The ability of RSB11-HI to modulate β-glucuronidase and estradiol levels expands its relevance beyond inflammation. β-Glucuronidase is essential for estrogen deconjugation and recirculation; its dysregulation has been linked to estrogen deficiency and reproductive dysfunction [6,38]. By enhancing this pathway, RSB11-HI may restore estrogenic tone in disorders such as PCOS, menopause-associated bone loss, and estrogen-deficiency mood disorders. These conditions are named only to indicate where the pathway is thought to operate and not as indications for which the present data provide evidence. The observations reported here come from a two-compartment conditioned-medium model built on immortalized cell lines with a single hormonal readout, and clinical inference from them would be premature. However, the integration of endocrine modulation with anti-inflammatory activity differentiates RSB11-HI from conventional postbiotics, suggesting plausible unique value in women’s health and broader metabolic diseases.

Our cytokine analyses confirmed that RSB11-HI exerts broad anti-inflammatory effects [34,39]. IL-6 and IL-1β, both pivotal drivers of acute inflammation and chronic disease, were robustly induced by LPS in epithelial, hepatic, renal, and osteoblast-like cells. Treatment with RSB11-HI consistently reduced both intracellular and secreted cytokine levels, with the strongest suppression observed in MG63 and A498 models. These findings complement the previously demonstrated inhibition of TNF-α and MMP-9, together establishing that RSB11-HI targets multiple inflammatory nodes [34,39]. The ability to concurrently suppress proteases, cytokines, and upstream drivers of inflammation positions RSB11-HI as a broad-spectrum immunomodulator with potential utility in conditions such as inflammatory bowel disease, arthritis, pulmonary fibrosis, and cancer, where these mediators are central to pathology. The superior IL-6 suppression observed with RSB11-HI in MG63 cells may potentially reflect enhanced exposure of structural cell wall components such as peptidoglycan, teichoic acids, and surface-layer proteins following heat inactivation, which could render these immunomodulatory ligands more accessible to host pattern recognition receptors. It is plausible that these stable structural elements engage TLR2/NF-κB signaling pathways more efficiently in the absence of active bacterial interference, though the precise mechanism warrants further investigation. Our previously published RNA-seq data [32], demonstrates that *Lactiplantibacillus plantarum* and other strains robustly downregulate the NF-kB signaling pathway in Human Bronchial Epithelial Cells (HBEC) at a Fold Change ≥ 2 (*p* < 0.05). Additionally, our data in the same publication (Figure 3) shows that *Lactiplantibacillus* cell wall components—including teichoic acid, peptidoglycan, polysaccharide, and S-layer significantly reduce MMP-9 levels (Fold Change ≥ 5, *n* = 6, *p* < 0.001) in HBEC exposed to *E. coli*. While these findings were obtained using the live RSB11 strain, future studies will compare these results to the heat-inactivated RSB11-HI strain in multi-organ settings.

A major barrier to probiotic therapy is the requirement for microbial viability [40,41]. Live probiotics are prone to loss of efficacy during storage and distribution, necessitate strict cold-chain logistics, and can pose safety concerns in vulnerable or immuno-compromised populations [14,42]. Our findings that RSB11-HI is fully active and, in some cases, more potent than its live form demonstrate that heat inactivation does not compromise function but instead provides distinct advantages. By eliminating the dependence on microbial survival, RSB11-HI allows for stable formulations with extended shelf-life, reduced manufacturing costs, and compatibility with diverse delivery platforms (capsules, powders, beverages, or topicals). Furthermore, safety is inherently improved, removing concerns of uncontrolled replication, horizontal gene transfer, or sepsis in high-risk patients [43,44]. Thus, RSB11-HI represents a paradigm shift from “live-dependent” probiotics to next-generation postbiotics optimized for safety, stability, and scalability.

One of the most striking findings of this study is the ability of RSB11-HI to modulate β-glucuronidase levels and estrogen metabolism along the gut–ovarian axis. β-Glucuronidase is a key microbial enzyme involved in the enterohepatic recirculation of estrogens [45]. By enhancing β-glucuronidase levels, RSB11-HI increased estrogen bioavailability in ovary–gut co-culture models, leading to significantly elevated estradiol levels compared to control or live RSB11 treatment. This dual action, dampening inflammation while restoring estrogen signaling, suggests that RSB11-HI may influence a broader network of host–microbiome interactions that extend beyond classical immune pathways. Dysregulation of estrogen metabolism is implicated in reproductive health disorders, osteoporosis, cardiovascular disease, and hormone-driven cancers [46]. Thus, the ability of RSB11-HI to modulate both immune and hormonal pathways positions it uniquely as a therapeutic candidate at the intersection of inflammation and metabolism.

These observations belong to a substantial and growing body of work on heat-treated lactobacilli and are best read as an extension of it rather than as an isolated finding. Heat-killed preparations of *Lactiplantibacillus plantarum*, *Lacticaseibacillus rhamnosus* GG, and related strains have repeatedly been shown to suppress TNF-α, IL-6, and IL-1β in intestinal and airway epithelial models, in macrophage co-culture systems, and in animal models of colitis and airway inflammation, in several cases with efficacy equivalent to that of the corresponding live organism. The magnitude of cytokine suppression observed here, approximately 40–70% for TNF-α, falls within the range described for other heat-treated lactobacilli. The retention of activity after inactivation supports the prevailing view that much of the immunomodulatory effect resides in heat-stable cell-envelope structures rather than in secreted or viability-dependent metabolites. And the occasional superiority of the inactivated form, also reported for other strains, is compatible with greater accessibility of those structures after thermal treatment. What the present work adds is breadth rather than a new phenomenon. To our knowledge the equivalence of live and heat-inactivated preparations has not previously been assessed for a single strain across six organ-representative human cell models in parallel, and the coupling of that anti-inflammatory profile to β-glucuronidase and estradiol readouts in a gut–ovarian conditioned-medium model has not been described. The contribution therefore lies in the systematic cross-tissue comparison and in the endocrine readout, not in a claim that heat-inactivated lactobacilli are anti-inflammatory per se.

From a translational perspective, the retained activity and greater effects observed in selected contexts with RSB11-HI relative to the live form opens multiple therapeutic avenues [47]. The combined suppression of TNF-α, IL-6, and IL-1β mirrors the targets of several high-value biologics currently used in clinical practice, such as anti-TNF and anti-IL-6 therapies. Moreover, the capacity to enhance β-glucuronidase levels and estrogen availability introduces opportunities for application in women’s health, including conditions such as polycystic ovary syndrome (PCOS), infertility, menopause-related osteoporosis, and estrogen-linked mood disorders. The integration of immune dampening and hormonal regulation is especially relevant to diseases where chronic inflammation and endocrine imbalance converge, such as metabolic syndrome, autoimmune conditions, and hormone-driven cancers. The cell-type dependent variability in altered levels of inflammatory cytokines is an interesting phenomenon. Caco-2 gut epithelial cells are well known to exhibit LPS tolerance due to reduced TLR4 surface expression and constitutive microbial exposure, which attenuates their IL-6 and IL-1β responsiveness. In contrast, MG63 and A498 cells exhibit heightened TLR4/NF-κB responsiveness given their lack of chronic microbial exposure, making them more sensitive to both LPS stimulation and RSB11-HI-mediated suppression. This context-dependent response reflects the physiological heterogeneity of inflammatory signaling across organ systems.

Together the preservation of anti-inflammatory activity following heat inactivation, combined with the unique hormonal modulation of RSB11-HI, positions it for dual-action indications. Unlike biologics (e.g., anti-TNF, anti-IL-6 antibodies), which are costly and immunosuppressive, RSB11-HI represents a safe, orally deliverable, and cost-effective intervention [48,49]. Its dual targeting of inflammation and estrogen balance may be especially valuable in female-predominant diseases where endocrine dysfunction exacerbates immune activation (e.g., autoimmune thyroiditis, endometriosis, rheumatoid arthritis).

Several constraints bound the interpretation of this work. First, the study is conducted entirely in vitro and contains no primary cells, no animal model and no human data; the findings describe cellular responses under controlled challenge rather than therapeutic effect. Second, with the exception of 16HBE14o−, an SV40-immortalized line derived from normal bronchial epithelium, the models used are cancer-derived (Caco-2, BG-1, MG-63, A-498, HepG2) and carry altered signaling, proliferative and metabolic characteristics that may not reproduce the behavior of the corresponding primary tissue. Third, the gut–ovarian experiments rely on the unidirectional transfer of conditioned medium, which captures only the secretory output of the intestinal compartment and cannot reproduce the bidirectional, spatially organized and circulation-dependent communication implied by the term “axis”. Fourth, the rise in measured estradiol cannot be assigned to a single mechanism from these data; deconjugation of glucuronidated estrogens is the most parsimonious explanation given the concurrent increase in β-GUS, and de novo bacterial synthesis is improbable in a heat-inactivated preparation, but stimulation of endogenous steroidogenesis in BG-1 cells has not been excluded and would require substrate labelling or selective β-glucuronidase inhibition to resolve. Fifth, no RSB11 life comparator was included in the β-glucuronidase experiments, leaving the specificity of that effect to the inactivated form open. Sixth, the heat-inactivated preparation is not sterile: the lot used here carried 9000 CFU/g of residual culturable organisms, approximately 27 CFU/mL at the working concentration, and culture-based enumeration does not in any case exclude a viable-but-non-culturable fraction. Complementary assessment by flow cytometry with membrane-integrity staining or by propidium monoazide qPCR would strengthen this. A further constraint is that the commercial ingredient contains microcrystalline cellulose as a carrier, and its documentation does not specify whether lysates, fermentation supernatant, or secreted metabolites are also present, so the active fraction within the preparation has not been resolved. Finally, the mechanistic account offered above, which attributes activity to heat-stable cell envelope components acting through TLR2/NF-κB, is an inference drawn from the pattern of results and from our earlier work on purified cell wall fractions; it was not tested directly here by receptor blockade or pathway readout and is presented as a hypothesis rather than as a finding. Addressing these points through primary cell and in vivo studies, direct mechanistic interrogation, and side-by-side live and inactivated comparisons across all endpoints forms the natural next stage of this work.

This study, while comprehensive across multiple epithelial and non-epithelial in vitro models, remains limited to cell culture systems. Although these models capture key aspects of inflammatory and hormonal signaling, they cannot fully recapitulate the systemic complexity of host–microbiome interactions in vivo. Future validation in animal models and clinical cohorts will be vital to confirm the translational relevance of RSB11-HI, particularly in the context of enterohepatic circulation and endocrine cross-talk. Moreover, expanding analyses to include long-term dosing paradigms, microbiome compositional shifts, and tissue-specific hormone receptor activation could further strengthen mechanistic insight. Consistent with these findings, supplementation with purified bacterial cell wall components such as teichoic acid, peptidoglycan, polysaccharide, and S-layer, each independently attenuated MMP-9 expression in *E. coli*-challenged bronchial epithelial cells [32]. This suggests that structural components of Lactobacillus strains contribute significantly to the anti-inflammatory activity of RSB11-HI. Such results further support the hypothesis that postbiotic efficacy arises not solely from secreted metabolites but also from stable, cell-associated molecular patterns capable of modulating host immune signaling. The strong and consistent effects observed across multiple independent cell models provide a robust foundation for advancing RSB11-HI into preclinical development, supporting its potential as a dual-action postbiotic with both anti-inflammatory and endocrine-modulatory benefits. While the use of established cell lines ensured standardization and reproducibility across organ models, we acknowledge that the reliance on cancer-derived cell lines may not fully recapitulate primary tissue physiology, and future validation in primary cell systems will be an important next step.

## 5. Conclusion

Collectively, these findings establish RSB11 as a versatile biotic platform with robust biological activity in both its live and heat-inactivated forms. Across the six organ-representative models, LPS or *E. coli* challenge raised *MMP-9* mRNA approximately two- to three-fold and both RSB11 life and RSB11-HI returned it to near-baseline, TNF-α fell by roughly 40–70% depending on the model, IL-6 and IL-1β were suppressed most strongly in MG-63 and A-498 cells, with IL-1β in A-498 cells restored to near-control values, while Caco-2 and BG-1 cells showed limited intracellular IL-6 responses consistent with the reduced TLR4 responsiveness of gut epithelium. Neither preparation perturbed basal MMP-9 expression in the absence of challenge. In the gut–ovarian conditioned-medium model, RSB11-HI approximately doubled β-glucuronidase levels in gut supernatants and raised estradiol roughly three-fold relative to the control, although without a live comparator for the β-glucuronidase measurement. The preservation of broad anti-inflammatory effects following heat inactivation demonstrates that the functional potential of RSB11 is not dependent on microbial viability, while the additional effects of RSB11-HI on β-glucuronidase levels and estrogen-related responses further expand its functional profile. Together, the complementary potential of live RSB11 and RSB11-HI provides opportunities to tailor their use across different formulations and applications, supporting continued development of RSB11 as both a probiotic and postbiotic strategy for modulating inflammation and host–microbiome interactions.

## Figures and Tables

**Figure 1:**
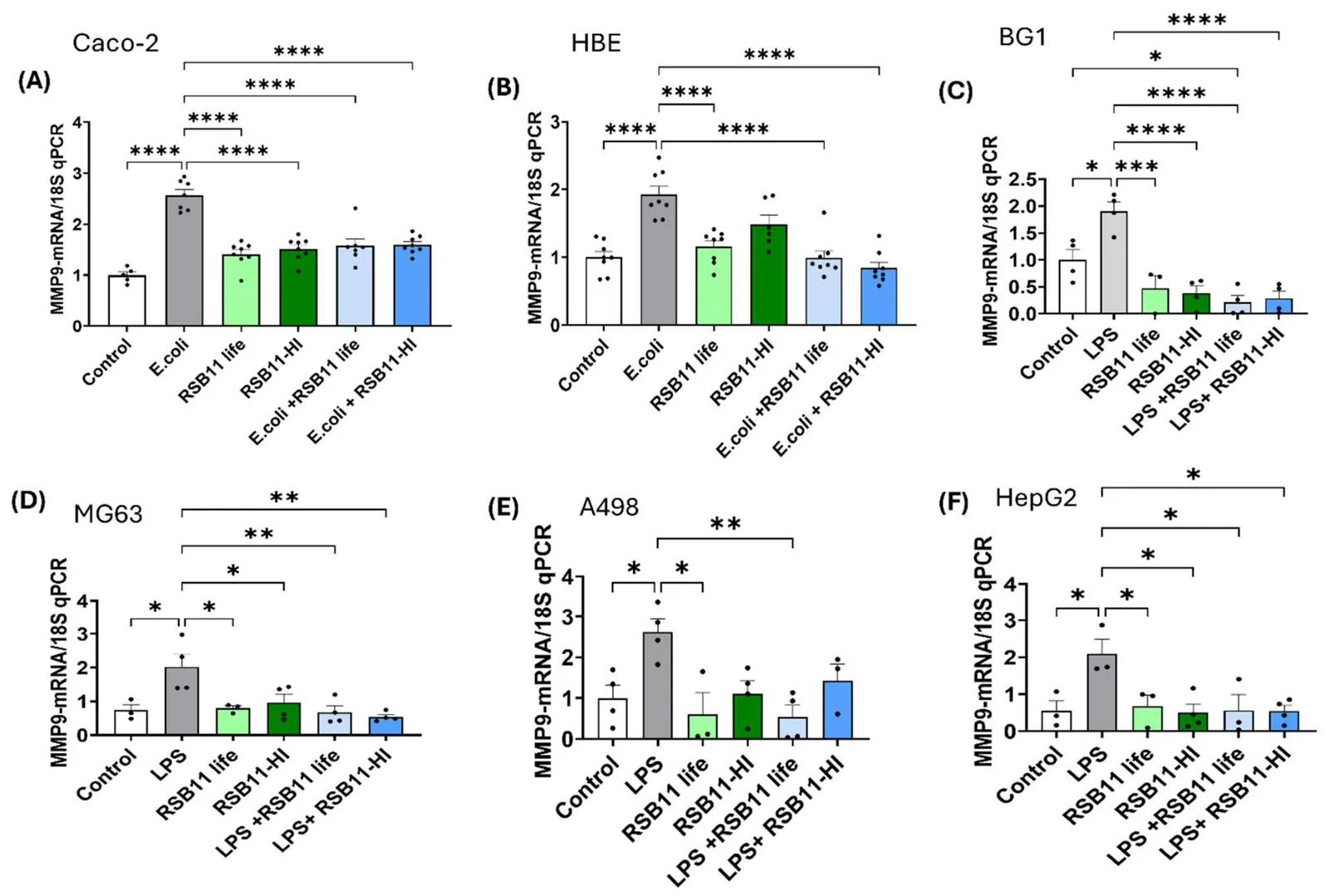
RSB11 life and RSB11-HI suppress MMP-9 expression across multiple cell models. Quantitative PCR analysis of *MMP-9* mRNA expression in (**A**) Caco-2 (gut), (**B**) HBE (lung), (**C**) BG1 (ovary), (**D**) MG-63 (bone), (**E**) A-498 (kidney), and (**F**) HepG2 (liver) cells following stimulation with LPS (1 µg/mL, 4 h) or *E. coli* (10^7^ CFU/mL). Both live (RSB11 life) and heat-inactivated (RSB11-HI) formulations (3 mg/mL) significantly suppressed MMP-9 induction to near-baseline levels, with no significant difference between the two forms. Neither RSB11 life nor RSB11-HI altered basal MMP-9 expression in the absence of inflammatory challenge. Data were presented as mean ± SEM; *n* = 3 independent experiments. *p* < 0.05, one-way ANOVA with Tukey’s post hoc test. * *p* < 0.05; ** *p* < 0.01; *** *p* < 0.001; **** *p* < 0.0001.

**Figure 2:**
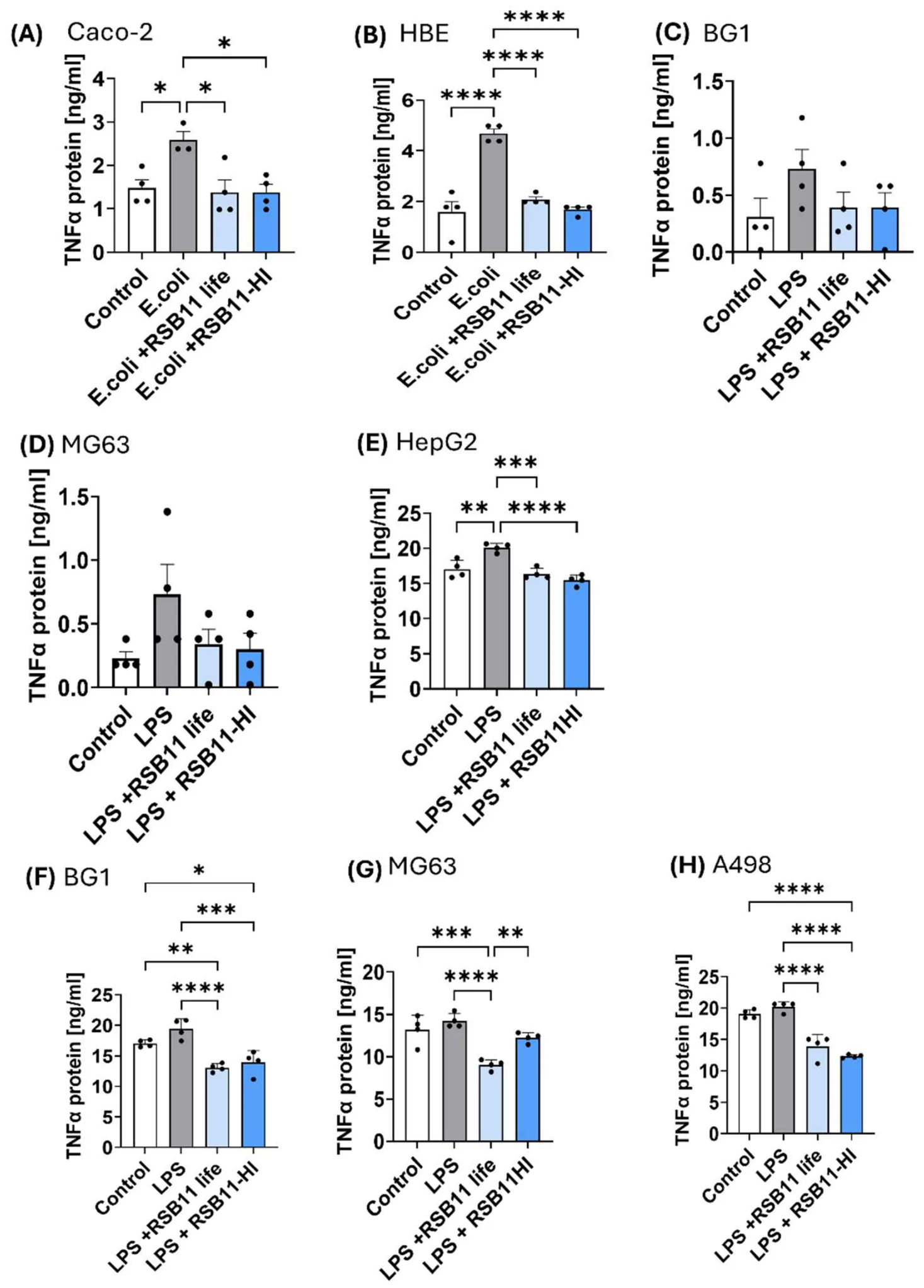
RSB11 life and RSB11-HI reduce TNF-α induction across multiple cell models. ELISA measurement of TNF-α levels in cell lysates and supernatants from epithelial (**A**–**C**,**F**: Caco-2, HBE, BG1) and non-epithelial (**D**–**H**: HepG2, A-498, MG-63) cell models following LPS or *E. coli* challenge. Both RSB11 life and RSB11-HI significantly reduced TNF-α secretion (40–70% reduction across models), with no significant difference between formulations. The retained activity of the live and heat-inactivated preparations highlights the robust anti-inflammatory activity of RSB11 independent of microbial viability. Data shown as mean ± SEM; *n* = 3 independent experiments. *p* < 0.05, one-way ANOVA with Tukey’s post hoc test. * *p* < 0.05; ** *p* < 0.01; *** *p* < 0.001; **** *p* < 0.0001.

**Figure 3:**
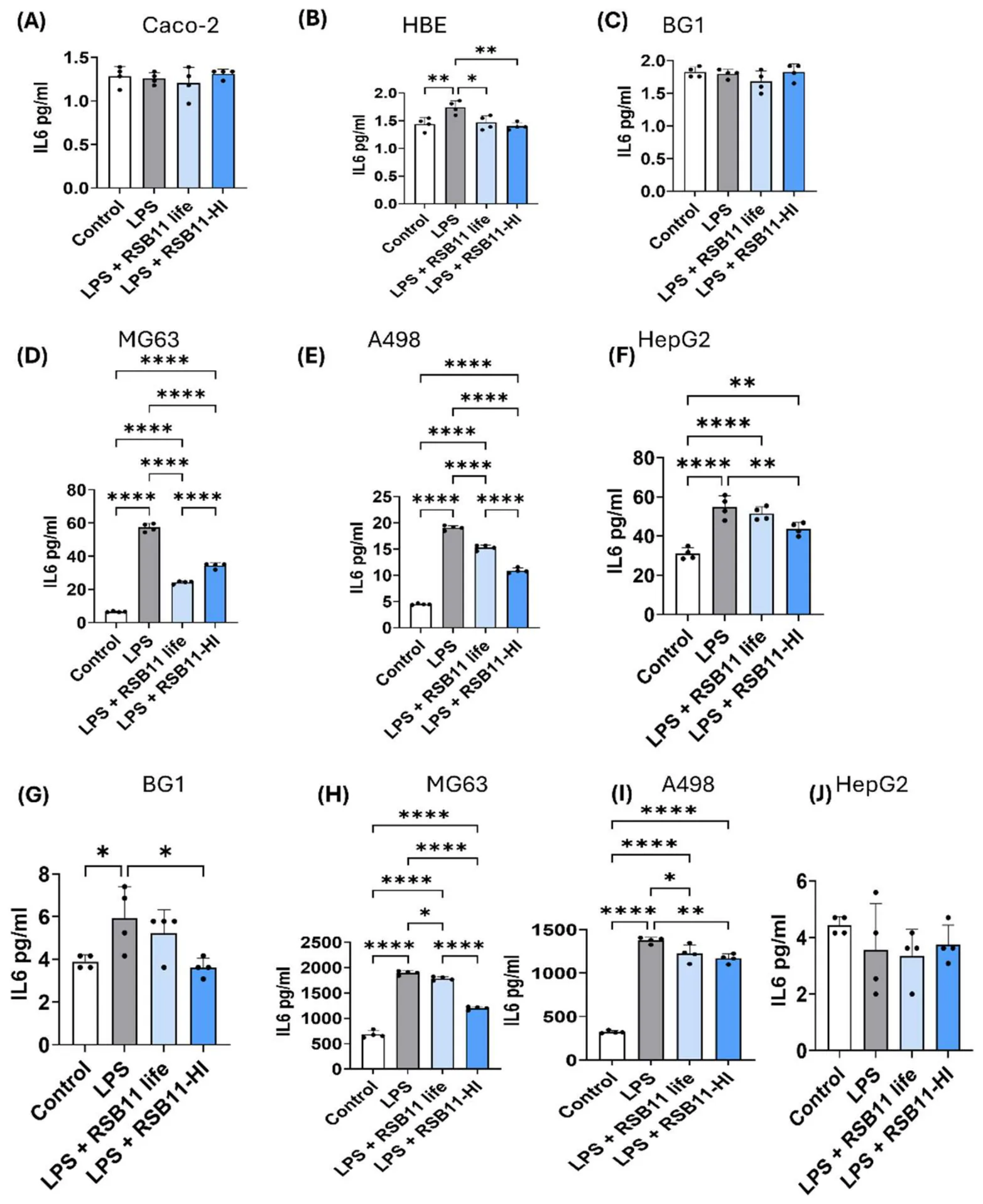
RSB11 life and RSB11-HI suppress IL-6 in (**A**–**F**) cell pellets and (**G**–**J**) supernatants. ELISA quantification of IL-6 levels in Caco-2, HBE, BG1, MG63, A498, and HepG2 cells. LPS stimulation significantly increased IL-6 expression across multiple cell types. RSB11 life and RSB11-HI significantly reduced IL-6 levels in HBE, MG63, and A498 cells. (**A**–**F**) intracellularly and in (**G**–**J**) supernatants, while little effect was observed in Caco-2 and BG1 cells. RSB11-HI produced stronger suppression than RSB11 life in MG63 supernatants. Data are mean ± SEM, *n* = 3–4, with *p* < 0.05, *p* < 0.01, *p* < 0.001. * *p* < 0.05; ** *p* < 0.01; **** *p* < 0.0001.

**Figure 4:**
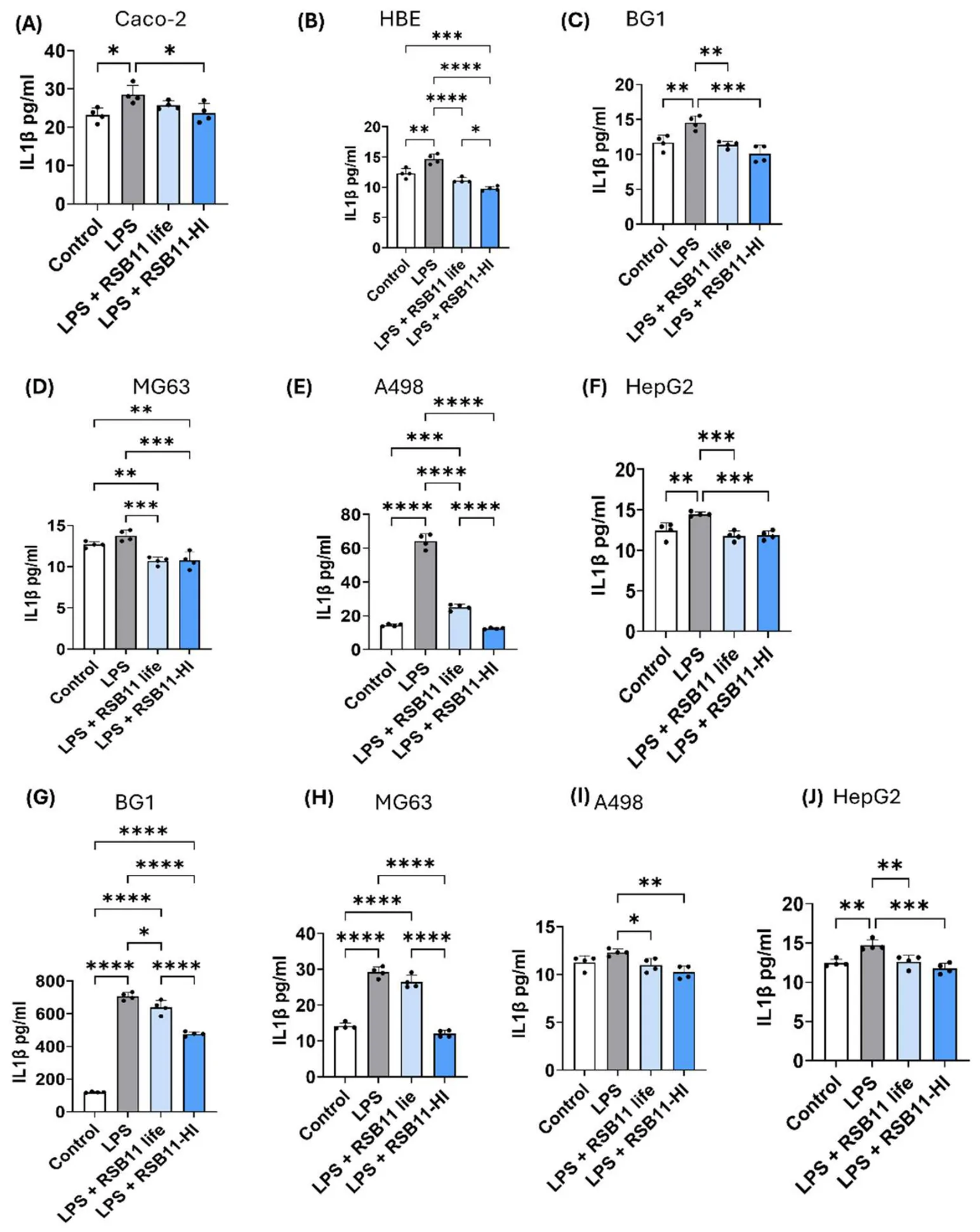
RSB11 life and RSB11-HI attenuate IL-1β in (**A**–**F**) cell pellets and (**G**–**J**) supernatants. ELISA measurement of IL-1β levels in Caco-2, HBE, BG1, MG63, A498, and HepG2 cells. LPS markedly increased IL-1β expression and secretion, in which RSB11 life and RSB11-HI significantly reduced in HBE, MG63, A498, and HepG2 cells. A498 cells showed the strongest suppression, with IL-1β nearly restored to control levels. Minimal effects were observed in Caco-2 cells, while BG1 cells showed significant reductions. RSB11-HI frequently demonstrated slightly stronger suppression than RSB11 life. Data are presented as mean ± SEM; *n* = 3 independent experiments. * *p* < 0.05; ** *p* < 0.01; *** *p* < 0.001; **** *p* < 0.0001.

**Figure 5:**
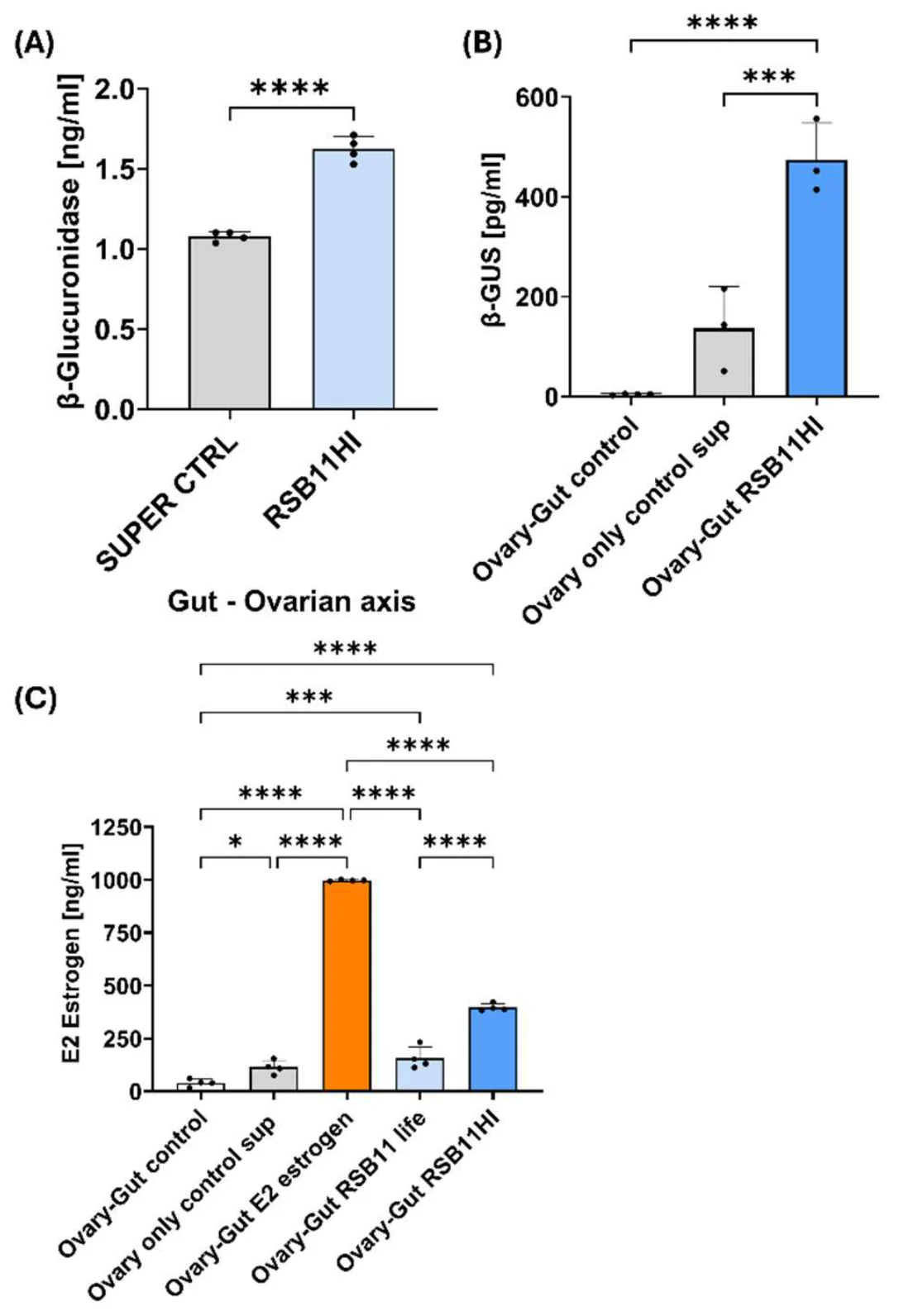
RSB11-HI enhances β-glucuronidase levels and estrogen production in gut–ovarian axis models. (**A**) β-glucuronidase (β-GUS) levels in gut supernatants were significantly increased following RSB11-HI treatment compared to supernatant controls. (**B**) In ovary–gut co-culture systems, RSB11-HI significantly upregulated β-GUS levels relative to untreated controls. (**C**) Estradiol (E2) levels were quantified across ovary–gut conditions. RSB11-HI markedly increased E2 levels compared to controls and RSB11 life, while exogenous E2 supplementation produced the highest levels. Data are presented as mean ± SEM; *n* = 3–4. *p* < 0.05; *p* < 0.001; *p* < 0.0001. * *p* < 0.05; *** *p* < 0.001; **** *p* < 0.0001.

**Table 1:** Cell lines and study design.

Organs	Cell Lines	qPCR	ELISA
Gut	Caco-2	MMP9	TNFα, IL-6, IL-1β
Lung	HBE	MMP9	TNFα, IL-6, IL-1β
Ovary	BG1	MMP9	TNFα, IL-6, IL-1β
Bone	MG63	MMP9	TNFα, IL-6, IL-1β
Liver	HepG2	MMP9	TNFα, IL-6, IL-1β
Kidney	A498	MMP9	TNFα, IL-6, IL-1β

## Data Availability

The original data supporting the conclusions of this article are included within the article and its Supplementary Materials. Further inquiries may be directed to the corresponding author.

## Supplementary Materials

The following supporting information can be downloaded at: https://www.mdpi.com/article/10.3390/cells15191736/s1, Supplementary File S1: Certificate of analysis and heat-inactivation information for RSB11-HI.

## Abbreviations

BPD	Bronchopulmonary dysplasia
HI	heat inactivated
COPD	Chronic obstructive pulmonary disease
TNF-α	Tumor necrosis factor-α
IL-6	Interleukin-6
IL-1β	Interleukin-1β
MMP-9	Matrix metalloproteinase-9
