# Supplementary material for: Heat-Inactivated Lactiplantibacillus plantarum (RSB11) as a Multifunctional Postbiotic: Anti-Inflammatory Effects Compared with Live RSB11 Across Multiple Human Cell Models
Source: Cells. 2026 Sep 23;15(19):1736. doi: 10.3390/cells15191736 (PMC13664586; doi:10.3390/cells15191736)
Supplement: Supplementary file 1 [file cells-15-01736-s001.zip › cells-4532467-supplementary.pdf]

Supplementary File S1

Functional Equivalence of Heat-Inactivated (HI) and Live Probiotic RSB11 in Suppressing Inflammation: Expanding Formulation and Application Potential

Teodora Nicola[†1], Tanmaya Madhvacharyula[†1], Ajay Ashok[†2], Aryan Mandot[1], Iman Abdelgawad[1], Ryaan Singh[1], Kara Siedman[1], Youfeng Yang[1], Namasivayam Ambalavanan[1], Charitharth Vivek Lal [3,4,5] 🖂.

Affiliations

- [1]Division of Neonatology, Department of Pediatrics, University of Alabama at Birmingham, Birmingham, AL, USA.
- [2]Schepens Eye Research Institute of Massachusetts Eye and Ear, Harvard Medical School, Boston, MA 02114, USA.
- [3]Division of Neonatology, Department of Pediatrics, University of Alabama at Birmingham, Birmingham, AL, USA. clal@uabmc.edu 🖂.
- [4]Lung Health Center, University of Alabama at Birmingham, Birmingham, AL, USA. clal@uabmc.edu.
- [5]Marnix Heersink Institute of Biomedical Innovation, University of Alabama at Birmingham, Birmingham, AL, USA. clal@uabmc.edu
- †These authors contributed equally to this work and share first authorship
- 🖂 Corresponding author

Availability of the Heat-Inactivated Postbiotic (RSB11-HI)

The life and heat-inactivated *Lactiplantibacillus plantarum* RSB11 strain was kindly provided by Resbiotic Nutrition Inc. (resbiotic.com). The freeze-dried powders, both live and heat-inactivated, were stored at -80°C to ensure the long-term stability of the biological material. In this study, the term “heat-inactivated” refers to a heat-treated preparation produced under controlled thermal conditions to ensure safety and functional stability, with process validation currently being finalized by the manufacturer. In short, steam injection into the fermenter was carried out at >80°C for 45 min. This is an established standard parameter for heat mediated inactivation[1]. Upon completion of the batch, the cell microscopy test passed the specifications for the heat inactivated material. The ‘Certificate of Analysis’ is provided below:

CULTURES SUPPORTING LIFE

CERTIFICATE OF ANALYSIS

PRODUCT CODE	PRODUCT DESCRIPTION
K001142	L. plantarum RSB11 HI 400B - Resbiotic 1 kg

FINISHED LOT	MIX LOT	BEST BEFORE	PRODUCTION DATE
FPU2500430	MXU2500189	07/2026	07/31/2025

MICROBIOLOGICAL PARAMETERS	SPECIFICATION	RESULT	METHOD
Inactivated Cell Count	≥ 4.00E+11 CFU/g	4.60E+11 CFU/g	QC-AM-016
Viable Cell Count (HI)	< 100,000 CFU/g	400 CFU/g	QC-AM-001
B. cereus	< 100 CFU/g	Conforms	FDA BAM CH. 14
E. coli	Not Detected in 10g	Conforms	USP <2022>
Listeria monocytogenes	Not Detected in 25g	Conforms	FDA BAM CH. 10
Salmonella	Not Detected in 25g	Conforms	USP <2022>
Staph. aureus	Not Detected in 10g	Conforms	USP <2022>
Enterobacteriaceae (Bile-Tolerant Gram Negative)	< 10 MPN/g	Conforms	USP <2021>
Non lactic acid bacteria	< 5,000 CFU/g	Conforms	QC-AM-005
Yeast/Mold	< 10 CFU/g	Conforms	QC-AM-009
Visual Inspection	White to tan, free flowing powder	Conforms	QC-AM-007
Water Activity	< 0.150 Aw	0.037 Aw	QC-AM-006

RELEASED

Date: 7/31/2025

Quality Department

Deb Day

Reference:

1. Wang, Xiaobing, et al. "Effect of Direct Steam Infusion Technology on the microorganism sterilization, Heat Intensity Indicators, and nutrients of Milk." *Applied Food Research* (2025): 101457.
